# Decision Making With an Alternative Mindset in an Online Shopping Environment: Identifying User Intentions Toward Facebook-Commerce

**DOI:** 10.3389/fpsyg.2022.848931

**Published:** 2022-03-07

**Authors:** Jian Wang, Fakhar Shahzad, Imran Khan, Abdul Waheed Siyal

**Affiliations:** ^1^College of Economics and Management, Zhengzhou University of Light Industry, Zhengzhou, China; ^2^School of Management, Jiangsu University, Zhenjiang, China; ^3^Department of Management Science, The Islamia University of Bahawalpur, Bahawalnagar, Pakistan; ^4^Department of Business Administration, ILMA University, Karachi, Pakistan

**Keywords:** trust, f-commerce, perceived value, gender, eWOM

## Abstract

Considering the current global trend in the digital economy, Facebook commerce (f-commerce) is an indispensable component of today’s digital commerce. The purpose of this research is to test the proposed model to validate current theories that evaluate the relationships between online trust and customer f-commerce usage intentions. It has also been proposed to improve usage intention by integrating the relationship between perceived value and trust in electronic commerce (e-commerce). The data was gathered using a structured questionnaire and analyzed with structural equation modeling (SEM; *n* = 435). In this study, gender has been discussed as a moderating factor. The findings describe the positive relationships that exist between antecedents of trust and user intentions toward f-commerce. Furthermore, according to the findings of the study, females are more concerned than males about developing their intentions to utilize f-commerce for online shopping. Theoretical and practical implications are also presented in this study.

## Introduction

Online retailers have begun to add components to their online stores in the present digital age, where Internet users can communicate with each other and vendors, while others stream electronic commerce (e-commerce) through Facebook, one of the world’s largest social networks (Wingreen et al., 2019; Chiu, 2020; Busalim et al., 2021). Meanwhile, the Facebook platform helps communicate and exchange information to develop synergies between consumers and retailers (Gibreel et al., 2018). Even though Facebook is a powerful social media tool, potential customers spend much time chatting before purchasing Facebook commerce (f-commerce). This helps build their trust in the product, electronic retailer/online retailer (e-retailer), and on the f-commerce platform (Liébana-Cabanillas and Alonso-Dos-Santos, 2017). It remains far discovered that 96% of Fortune-500 professional stores also use the Facebook platform. Vendor Facebook pages have empowered approximately 86% of customers to trade products and services, specifically in the Asian region (Leong et al., 2018). Likewise, the social function of Facebook for marketing enables consumers to share and recommend products and e-retailer to each other. As per the recent report of Chaffey (2022), 57.6% of the world’s population uses social media including Facebook. Average usage is 2 h and 27 min per day. The e-commerce industry in Pakistan is rapidly emerging and has the potential to strengthen the country’s economy. Existing ICT infrastructure connects remote areas to the mainstream (Shahzad et al., 2020). Building a Facebook page is much simpler than building a website from scratch and can be done in minutes with no financial outlay or technical expertise, beneficial for the firms working in developing countries like Pakistan. F-commerce in Pakistan is still in its infancy, and it is important to identify the key aspects of its successful adoption.

At the beginning of a new relationship, a disposition to trust is the main factor affecting their trust before evaluating whether the organization or person can be trusted or not (Mahad et al., 2018). In line with the trust theory, interaction with social media (i.e., Facebook) will transfer trust from one known mean to an unknown mean and change trust, leading toward usage intentions (Belanche et al., 2014). In f-commerce, trust becomes a prerequisite for building a consumer relationship (Alonso-Dos-Santos et al., 2019; Akram et al., 2020; Ashraf et al., 2021). Although f-commerce is an emerging concept, more exploration is needed to identify several factors associated with the development of trust because uncertainty in the environment, particularly in developing countries, reduces consumer trust (Chen and Wang, 2016). Moreover, the use of the f-commerce is quite different in developing nations and posited that due to dissimilar intentions and levels of trust. Therefore, current research seeks to address this gap by inspecting the key antecedents of trust in measuring the f-commerce usage intention, particularly in developing countries. Moreover, a more concise acquaintance of the crucial precursors of trust in contributing to the development of consumers’ f-commerce usage intention is a promising research area that further requires examination.

Meanwhile, in recent years, consumer perceived value has played a crucial role in forecasting consumer buying intentions, obtaining a competitive advantage, and influencing relationship management (Yang and Zeng, 2018; Wang and Zhang, 2019; Riaz et al., 2020). The study of Ponte et al. (2015) posits that consumer purchase intention using online shopping platforms has been influenced by perceived value. However, while shopping online, consumers can easily discover substitutes, so establishing an enduring relation poses a novel problematic wrangle for e-commerce and f-commerce. Perceived value contributes to social-commerce trustworthiness by reducing the consumer’s intentions to seek substitute service providers (Han and Windsor, 2011). Meanwhile, perceived value is crucial to developing trust in an online shopping environment; prior research has studied a consumer’s value in traditional settings and is less focused on studying online consumer behavior (Akram et al., 2017; Gan and Wang, 2017). Thus, the second objective of our investigation is to probe the moderating role of perceived value on the association of trust and f-commerce usage intention.

Furthermore, it is also essential to consider demographical factors, especially gender, where the research investigates behavioral intention (Dutta et al., 2018). Many studies concluded that gender differences vary in the diverse nature of relations and should be studied to get the actual strength of relationships about gender (Van Slyke et al., 2010; Tan et al., 2014; Escobar-Rodríguez et al., 2017; Mercanti-Guérin, 2021). A positive connection between the predictors and the dependent variable might be negative when the study considers gender as a moderator, so without studying moderating effects of gender, the research may present erroneous results in different fields (Durndell and Haag, 2002; Shahbaz et al., 2019). Therefore, the less focus on gender differences in the social commerce field motivates the authors also to investigate this important aspect in f-commerce.

Thus, we focus on the following key research questions.

1. What is the role of crucial factors to develop trust in f-commerce that influence consumers’ f-commerce usage intention?
2. How does the perceived value boost consumer trust in developing their intention to use f-commerce?
3. How do gender differences influence the relationships among different variables?

## Literature Underpinning

The behavior of the transaction associates might not be fettered in a business transaction. This is particularly valid during circumstances where visual, nonverbal, or further communicative gestures are notably absent (Reichheld and Schefter, 2000). Individuals expect that the other party involved in the business activities and the transaction would act responsibly (Gefen et al., 2003). Moreover, Chen and Shen (2015) analyzed trust on the way to online society and trust toward individuals in the system as essential elements of social sharing and social shopping. Prior studies analyzed that trust has a critical effect on consumer buying behavior (Farivar et al., 2017; Hajli et al., 2017; Akram et al., 2018; Sanyala and Hisamb, 2019). Accordingly, trust is often grounded in consumers’ previous purchase experience and ongoing relationship to their social community instead of transaction experiences, expressing inner state satisfaction (Chow and Shi, 2014; Wingreen et al., 2019). Sketching on the trust theory, we have developed a hypothetical model that highlights the crucial factors of trust demonstrated as the impact of f-commerce usage intentions (Hsu, 2019). Trust transfer is an intellectual procedure in which an individual may have transferred from one recognizable milieu to another unique milieu (Wang and Ellinger, 2011; Liu et al., 2018; Shahzad et al., 2019; Sharma et al., 2019; Lonardi et al., 2021). Recent studies explored the effect of trust on customer buying behavior in social commerce research using a sole component of trust or a solitary authorization (Kim and Park, 2013; Hajli et al., 2017; Dabbous et al., 2020). Investigating the role of trust in f-commerce is an immediate research area due to the dearth of prior studies in this field (Lu et al., 2016; Liébana-Cabanillas and Alonso-Dos-Santos, 2017; Al-adwan and Kokash, 2019). Therefore, this study examined theory-driven constructs of trust that influence individual decisions to take an active part in the use of the Facebook platform for their online purchases.

At the beginning of a new relationship, a disposition to trust is the main factor affecting their trust before evaluating whether the organization or person can be trusted or not (Mahad et al., 2018). The study of Schoorman et al. (2007) identified disposition to trust as “the extent to which a person tends to be dependent on others, in multiple situations, and among groups of people.” The study of Gefen (2000) empirically confirms prior non-empirical recommendations about the implicit impact of people’s disposition to trust on their early-stage trust in situations, where there is a dearth of extensive communication in social and organizational environments. Trust disposition is a personality-based trait, which enlightens why some of us tend to trust or distrust others (Wang et al., 2015). Similarly, under the current research settings, the disposition to trust is also essential for improving consumer trust in f-commerce. Besides the importance of disposition to trust, social network sites, e.g., Facebook, are often liable for their client’s security and privacy in developing the trust (Kim, 2013; Morgan Stanley, 2015; Chen and Wang, 2016). Moreover, from the prior theories of trust and technology adoption, it is proved that perceived security and privacy play a significant role in constructing individual trust toward the adoption of self-service technologies (Shin, 2010; Al-Sobhi and Weerakkody, 2011; Beldad et al., 2012; Dastan and Mesut, 2015; Ranaweera, 2016; Shahzad et al., 2019). In the area of social commerce, mainly in f-commerce, security and privacy need to be taken into consideration to create customer integrity and trustworthiness.

As social networking has become frequently available to the public, consumers have started using it as a potential source of information regarding corporations, products, brands, and services. Several e-retailers are currently using social technology and services to expand their businesses to get a competitive advantage (Zhou et al., 2013). Consumer attitudes are equally important to their e-retailers (Hajli et al., 2017). Consumers’ trust can be advanced based on the cognitive notion of e-retailers. Trusting every e-retailer is challenging these days. It is worthfully important to identify e-retailer loyalty while using digital platforms (Shahzad et al., 2021). Therefore, we incorporate trust in e-retailer in our research framework, which ultimately develops consumer trust in f-commerce and leads toward usage intentions. In addition to the previous concept, viral marketing turns out to be allied with electronic word-of-mouth (eWoM) all through electronic media. The consumers share information about social shopping sites such as Facebook; eWoM reviews and interactive suggestions play a noteworthy part in social and f-commerce transactions (Sami et al., 2016; Prahiawan et al., 2021). A social shopping site for selling goods is highly reliable if it has positive feedback and consumers view eWoM (Sharma et al., 2019; Xu et al., 2021).

Moreover, driven on the equity theory, perceived value has spoken to the adjustment between the value and assistance that gets by the user, and the costs, for example, time, financial, and psychological transaction costs, which caused by assessing, getting, and utilizing a product or service by the users (Komulainen et al., 2007; Hsin Chang and Wang, 2011). When seeing customers’ purchase decision is not handiest stricken by the objective value of products or services, but additionally *via* the perceived value of services or products. Meanwhile, the customer’s notion of organizational ethics positively influences the improvement of consumer trust, which causes purchase (Choi et al., 2013; Samudro et al., 2020). Therefore, we also investigate the moderation effect of consumer perceived value on the association between trust in f-commerce and f-commerce usage intentions. Prior studies proved that the influence degree of each factor on individual behavioral intention varies in males and females (Venkatesh et al., 2012; Mandari and Chong, 2018; Xie et al., 2021). The research of Sun and Zhang (2006) pointed out that men are more realistic and motivated by the need for achievement, while women are anxious and more vulnerable to the immediate environment when making decisions. Furthermore, Venkatesh et al. (2012) explained that women have a greater reliance on external assistance to accomplish their goals than men. However, scholars on the pitch of f-commerce adoption neglected this vital role of gender as moderating the relationship among variables. Therefore, we further examine the moderating role produced by gender in current study settings.

Based on the extensive literature evaluation, it is substantially proved that the integration of fundamental elements of the trust will lead to enhance the consumer intends to use the f-commerce. Therefore, the research gap is fulfilled by incorporating a couple of critical trust precursors, while estimating individual intention to use the f-commerce platform for online shopping as well as the moderating role of gender and perceived value.

## Research Model and Development of Hypotheses

**Figure 1:**
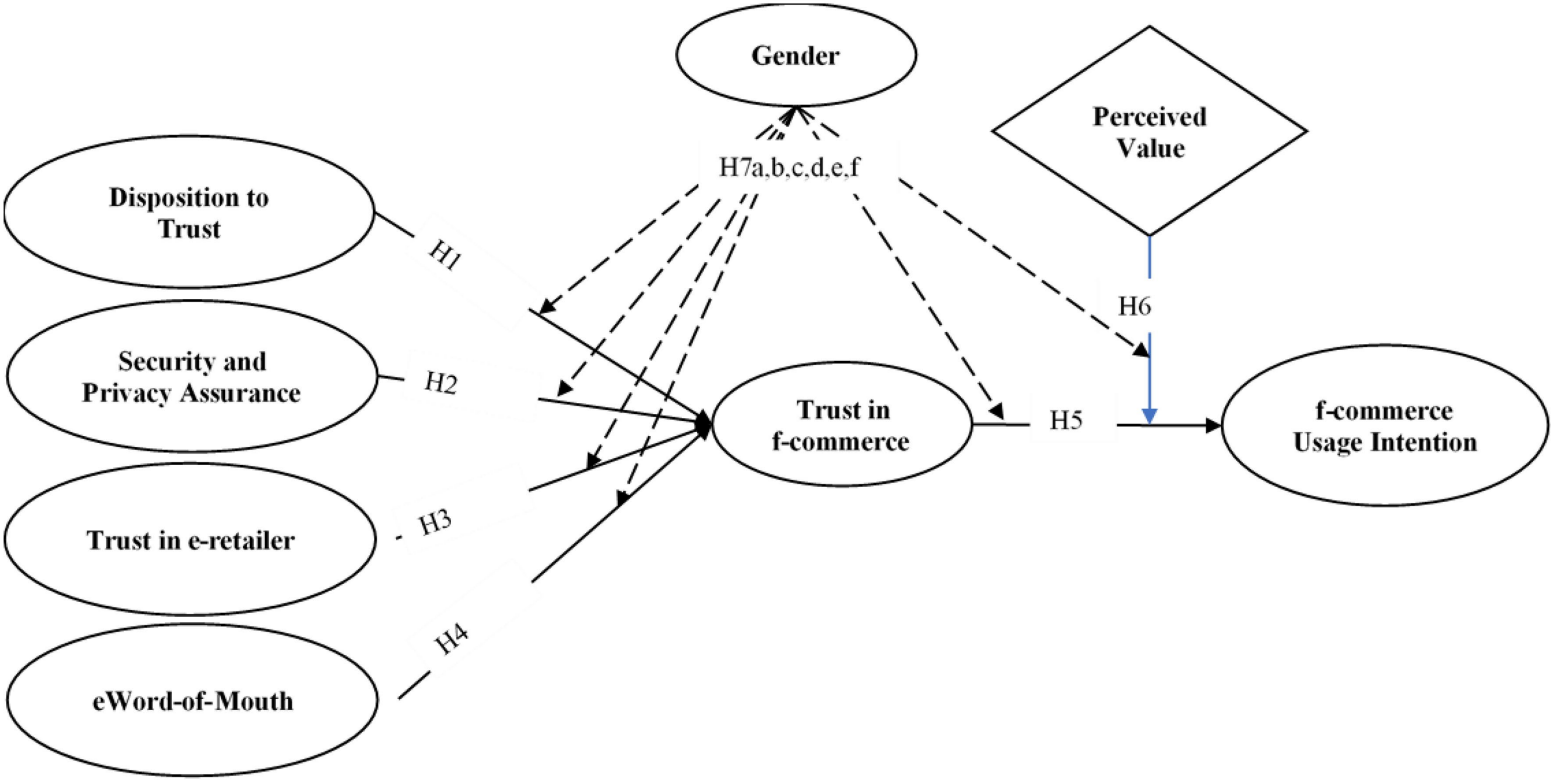
Proposed research model.

As described earlier, previous literature does not adequately support the development of trust in context to the development of f-commerce usage intention as a fast-growing aspect of social commerce, particularly in developing countries. Therefore, this research is going to establish an exhaustive insight from the literature related to trust, e-commerce, and social commerce adoption in conjunction with the consumer buying behavior with the attendant use of f-commerce. In this paper, Figure 1 demonstrates the conceptual framework of this paper to comprehend several important trust factors, which influence the intention of people to participate in f-commerce and ensure the moderating perspective of gender and perceived value.

### Disposition to Trust

Trust is perseverance shaped through a general disposition to trust and the process of continuous socialization. Disposition of trust refers to the inherent tendency to agree with others (Gefen, 2000). Individuals’ disposition to trust is not specific to distinctive entities/individuals but a constant characteristic of their persona, which determines how they see the credibility of other entities they come across (Escobar-Rodríguez et al., 2017; Sarkar et al., 2020). In preferred, trust is likewise the outcome of trust disposition. This disposition is, in particular, convincing while the trustor does not interact with a selected enterprise or individual on the unique issue (Gefen, 2000; Mahad et al., 2018). A study by Wang et al. (2015) revealed that the effect of disposition to trust on the digital service adoption intention had not been noted exceedingly within the social science literature. Conferred to the nature of f-commerce activities, any kind of interaction with the f-commerce platform is unlikely to have the same impact as a lifetime of acquired disposition (Sarkar et al., 2020). As a precursor to trust, the disposition to trust is highly efficient in the early stages of a relationship while both parties are unknown on the f-commerce platform. Therefore, we posit:

*H1:* Disposition to trust has a positive impact on developing trust in f-commerce.

### Security and Privacy Assurance

Consumers have been worried about information protection and the utilization of information by the supplier while engaged in online shopping (Fogel and Nehmad, 2008; Zafar et al., 2021). Although, consumers are also required to ensure personal and genuine information on their Facebook profiles (Brock et al., 2011). Along these lines, security and privacy issues, which have been firmly identified with trust in perceptions, are an unmistakable subject of discourse and assume a noteworthy part in the behavioral utilization of Facebook members (Proudfoot et al., 2018; Aslam et al., 2020). In the contentions described above, it ought to be accepted that trust in f-commerce can be improved by assuring consumer security and privacy. Shopping online itself is a risky activity, risk allied with susceptibilities about consequences, information security, and the concealed activities of the e-retailer (Zhang et al., 2013; Farivar et al., 2017; Sharma et al., 2019). Thus, this paper assumes that the increased level of security and privacy assurance in social commerce organizations will reduce the risk of data confidentiality and may lead to trust in f-commerce. Therefore, we hypothesized:

*H2:* Security and privacy assurance has a positive impact on developing trust in f-commerce.

### Trust in e-Retailer

Trust in e-retailer has been referred to as “the extent to which a consumer has confidence in the retailer’s consistency of trust in an online shopping environment” (Kaufman et al., 2006). The studies conceptualized three predominant variables of client trust in e-retailer: perceived benevolence, integrity, and competencies of e-retailer. *Benevolence* suggests that the e-retailer is keen on profit-making and working together in a manageable and palatable way for all concerned parties. The idea of *integrity* alludes to the consistency standards and authentic activities of the e-retailer. The last determining factor of trust in this setting is *competencies* or the e-retailer abilities and capabilities for working such e-business activities (Boone et al., 2006; Park et al., 2012). Many Facebook shops are associated with formally prevailing e-commerce vendors. Consumers feel that trusted e-retailers monitor personal information on social networks to a minimum extent and will not expose it to unauthorized users, which is why they trust them (Sharma et al., 2019; Wingreen et al., 2019; Fadhillah et al., 2021). In the current formation, it is anticipated that trust in the e-retailer is a core determining factor of trust levels in f-commerce.

*H3:* The trust in e-retailer has a positive impact on developing trust in f-commerce.

## eWord-of-Mouth

Before, when consumers required information, they usually moved to marketers, asked friends, and had discussions out in the community places like parks, markets, and shops (Fei, 2011; Alhidari et al., 2015). As explored by Kim and Park (2013), the effect of social commerce features such as word-of-mouth, recommendations, comments, and reviews, on/with trust in therefore prompting a consumer to intend to purchase. eWoM is defined as all word-of-mouth communications through computers, Internet media, e-mail, using online groups, and online portals, which enhances its uniqueness more conveniently and other anonymous characteristics (Alhidari et al., 2015; Akram et al., 2021). eWoM advises potential, real, or former consumers on constructive or adverse representations of the product or organization, making it accessible to a wide variety of individuals and research organizations through the Internet (Chu and Kim, 2011; Fei, 2011; Liébana-Cabanillas and Alonso-Dos-Santos, 2017). In line with the social presence theory, this assumes that eWoM has a positive association with trust, which contributes to a stronger sense of consumer social existence on the online platform. Such a platform is fascinated by information sharing-based communication about products (Awad and Ragowsky, 2008; Hajli et al., 2013). The statements from eWoM will directly influence consumer behavior and trust related to social-commerce adoption intentions (Sami et al., 2016; Liu et al., 2019; Goraya et al., 2021). A Filieri et al. (2018) endorses the definite link between trustworthiness and eWoM to investigate user intention about using an online system. For this reason, we hypothesized:

*H4:* The eWoM has a positive impact on developing trust in f-commerce.

### Trust and f-Commerce Usage Intention

The study of Liébana-Cabanillas and Alonso-Dos-Santos (2017) defines trust in two ways: (1) trust as credence, assurance, state of mind, or statement about additional gathering reliability; and (2) trust as intentional behavior or conduct of dependence and together with the susceptibility. Trust denotes security, sensation, and intent to depend on somebody or to some degree (Chung and Kwon, 2009). Several researchers explore the consequence of trust in usage intention and on purchase behavior in online settings. E-commerce and social commerce operations rely heavily on trust, which plays a key role in several corporate partnerships (Van Der Heijden et al., 2003; Brock et al., 2011). Trust has been theorized in many ways in the literature on social commerce and e-commerce. Prior studies posit that trust can enhance the consumer perception and intention to use online shopping, particularly by decreasing the social uncertainty and complexity engaged in multi-party exchanges and online transactions (Hajli et al., 2017; Alkhater et al., 2018; Liébana-Cabanillas et al., 2018; Sarkar et al., 2020). Concerning this study, trust in the f-commerce platform will ultimately influence its usage intentions. Thus, we formulate this hypothesis:

*H5:* Trust in f-commerce has a positive impact on developing consumers’ f-commerce usage intention.

### Moderating Role of Perceived Value

Perceived value is the outcome of checking consumers’ apparent interests and related sacrifices. Then again, perceived value also refers to a multi-dimensional factor through consumer value (Alshibly, 2014; Alonso-Dos-Santos et al., 2019). Meanwhile, consumer perceived value plays a considerable part in anticipating buying behavior, achieving sustainable competitive advantages, and influencing relationship management (Pakrou and Amir, 2016). A consumer’s perceived value has long been familiar with marketing research as a significant notion in measuring consumer intentions, influencing preferences, satisfaction, and trustworthiness (Yadav and Rahman, 2017). It is valuable for users to set up the product itself and the process of the online platform, channel and discovery, request, and receive the product (Hsin Chang and Wang, 2011; Kim and Park, 2013; Alshibly, 2014; Chen and Lin, 2015; Yuan et al., 2020).

Consumers can easily go for substitutions in an online purchasing atmosphere, so establishing long-lasting relations is a most worthwhile challenge for online firms (Hsin Chang and Wang, 2011; Chen and Lin, 2015). Furthermore, uncertainty in using a Facebook platform for buying will reduce the consumers’ intention to use f-commerce. Trust factors help to reduce uncertainty and enhance f-commerce usage intention, but it is highly significant to attain perceived value before using f-commerce (Ponte et al., 2015; Molinillo et al., 2021). In line with this study, grounded in previously mentioned literature, it has been concluded that perceived value is an inherent feature of the online shopping process, which is an impartial estimation of individual perception. This study assumes that perceived value intervenes in the association between trust and f-commerce usage intention, such that the greater the perceived value, the greater the consumer intends to use the f-commerce. Therefore, we bring forth the following:

*H6:* Perceived value moderates the relationship between f-commerce and f-commerce usage intention (increase in consumer perceived value boosts consumer trust in f-commerce and f-commerce usage intention).

### Moderating Role of Gender

Information technology (IT) is a necessary part of organizations, and gender differences should study, where IT involve in working (Shahbaz et al., 2020). It is imperative to study gender differences in social science research (Van Slyke et al., 2010; Dutta et al., 2018; Mandari and Chong, 2018; Wang et al., 2019). In different societies, gender skills and perceptions are different regarding usage intention, and it should be studied when investigating consumer buying behavior (Choudrie et al., 2010; Liébana-Cabanillas et al., 2018). Previous literature is evident that gender differences have moderating impacts in different research parameters (Hou et al., 2020). The study of Vekiri and Chronaki (2008) inspected the moderating impact of gender in online education and determined the female dominance in the relationship among predictor variables and intention to use online education. Another study demonstrates the impact of gender in technology adoption and concluded that gender differences are essential to research carried out in the adoption studies (Gefen and Straub, 2006). The study of Moon and Kim (2001) also studied the gender differences in web adoption and concluded the dominance of males.

Similarly, the study Khan et al. (2019) found a significant difference among gender in recognition and the use of e-health technologies. Unfortunately, in f-commerce adoption, the previous investigations put less focus on discussing gender differences. Few scholars focused on the gender differences in e-commerce (Hwang, 2010), m-commerce (Chong, 2013), and social commerce (Chen et al., 2018) and concluded that gender differences are the critical concern of the adoption of such type of commerce. Gender differences conceive at a higher rate while studying consumer buying behavior and the adoption of social platforms due to different preferences (Di Tommaso et al., 2020; Aguirre et al., 2021), especially in developing countries.

However, a more concise understanding of gender differences is required to build a further profound understanding of f-commerce adoption. Therefore, we also hypothesized that there are gender differences in the relationships of predictor variables and f-commerce usage intention.

*H7a:* Gender moderates the impact of disposition to trust in developing trust in f-commerce.

*H7b:* Gender moderates the impact of security and privacy assurance in developing trust in f-commerce.

*H7c:* Gender moderates the impact of trust in e-retailer in developing trust in f-commerce.

*H7d:* Gender moderates the impact of eWord-of-mouth in developing trust in f-commerce.

*H7e:* Gender moderates the relationship between trust in f-commerce and f-commerce usage intention.

*H7f:* Gender moderates the moderating impact of perceived value on the relationship between trust in f-commerce and f-commerce usage intention.

## Materials and Methods

### Research Instrument

In this study, the survey items have been adapted from prior studies. We have followed the suggestions of Fang et al. (2014) for question wordings when deciding the survey scale. Measurement items have been adapted from existing works and crammed according to the present study perspective, ensuring construct validity. The final questionnaire comprised 32 items (see in Appendix), which have been measured by using a seven-point Likert scale from 1 (Strongly disagree) to 7 (Strongly Agree). Various studies, for example (Preston and Colman, 2000; Bertot et al., 2010; Finstad, 2010; Krosnick and Presser, 2010), give compelling evidence and clarify the significance of employing the seven-point Likert scale as a survey tool. Moreover, demographic traits such as gender, age, and education have also been asked. To confirm content validity, the authors applied an expert evaluation method to polish the survey instrument. In this study, the age of the respondent was used as control variable to ensure that other variables are not influencing the empirical results because the prior literature posits that age contributes toward its usage intention (Lee et al., 2007; Fang et al., 2014; Liu et al., 2016).

### Sampling and Data Synthesis

This paper employed an online survey technique to collect required data because the target population included active users of Facebook and only those who have experience of shopping using f-commerce only in Pakistan. The online questionnaire was distributed using a link was sent to all members from the friend list on Facebook (friends from Pakistan). Friends are requested to perform the same distribution steps to maximize respondents’ participation by using snowball sampling. The participants were free to respond; we did not offer any incentive for participation. The studies suggested numerous strategies may be used in keeping with the nature of the research and target respondents for sample size (Kline, 2015). A study by Venkatesh and Bala (2008) suggested that for structural equation modeling (SEM) minimum sample of 200 respondents is reasonable for the analysis of results. This fieldwork for data collection began in November 2020 and finished in February 2021, with voluntary involvement. The original dataset contained 447 responses, which were reduced to 435 valid responses after the preliminary screening process (12 unengaged responses were removed).

### Data Analysis

For measuring and validating our research model, AMOS v24 was employed for the SEM, which is reflected as a useful tool to deal with confirmatory factor analysis (CFA) and structural modeling. Further, SEM takes into account the measurement errors in each measurement item to prevent biased conclusions (Alavifar et al., 2012). SEM also permits instant evaluation of multiple equations stipulated in the path model to produce useful information (Mandari and Chong, 2018). The SPSS v26 was employed to attain the initial statistics, reliability confirmation, and data screening.

## Results and Discussion

### Demographics of Participants

This section discusses the demographics of the attendees, such as gender, age, and education. Table 1 describes the overall composition of the demographic features of respondents. As per the results, 50.8% of the respondents were male, and the rest of 49.2% were female. 63.7% of the participants belong to the age group from 30 to 39, and 23% from 20 to 29. Most respondents are highly educated, of which 30.8% were graduates, and 22.5% were post-graduate. This composition describes that mostly young, educated respondents participated in this study, which may predict the apparent behavior of respondents regarding the use of f-commerce.

**Table 1:** Demographics of respondents.

Category		Frequency	Percentage (%)
Gender	Male	221	50.8
	Female	214	49.2
	Total	435	100.0
Age	20–29	100	23.0
	30–39	277	63.7
	40–49	39	9.0
	Over 50 years	19	4.4
	Total	435	100.0
Education	Undergraduate	75	17.2
	Graduate	134	30.8
	Post-graduate	98	22.5
	Others professional education	128	29.4
	Total	435	100.0

### Common Method Bias

When the required data were gathered from a single source and at the same point in time, the matter of common method bias (CMB) may peril the validity of research, can be tested *via* Harman’s single-factor analysis as proposed by Podsakoff et al. (2003, 2012). Several studies in social science research applied Harman’s single-factor analysis to test the CMB as employed in previous studies (Antonakis and House, 2014; Venkatesh et al., 2016; Sheikh et al., 2017; Cao et al., 2018; Liu et al., 2018; Shahzad et al., 2019). Therefore, consistent with these studies, the authors also employed the same to confirm the problem of CMB. The results from Harman’s single factor test categorized the items into seven factors, and the first factor describes only 35.6% variation, which is under the threshold point. Therefore, CMB was not incredibly alarming in our research.

### Measurement Model

Confirmatory factor analysis method has been applied to observe the measurement model as suggested by Hair et al. (1998, 2010), before analyzing the hypotheses to make sure that data reliability, construct validity, and instrument validity has no issues. The content validity was evaluated by reviewing the literature relevant to this study context and applying pilot testing of the survey instrument. Reliability was tested by applying Cronbach’s alpha, and the values range from 0.909 to 0.947, which confirms that the data is highly reliable, as shown in Table 2. Meanwhile, descriptive statistics such as “mean” and “SD” of the constructs have also shown in Table 2.

**Table 2:** Results of factor analysis, reliability, and descriptive statistics.

SR	Variables	Items	Loadings	Cronbach’s alpha	Mean	*SD*
1	Disposition to trust	DT1 DT2 DT3 DT4 DT5 DT6	0.837 0.848 0.855 0.810 0.854 0.860	0.947	5.20	0.989
2	Security and privacy assurance	SPA1 SPA2 SPA3 SPA4	0.829 0.852 0.809 0.833	0.912	4.76	0.799
3	Trust in e-retailer	TeR1 TeR2 TeR3 TeR4 TeR5	0.848 0.769 0.823 0.778 0.811	0.915	5.19	0.834
4	eWord of mouth	eWoM1 eWoM2 eWoM3 eWoM4	0.833 0.816 0.923 0.850	0.918	4.88	0.799
5	Trust in f-commerce	TR1 TR2 TR3 TR4 TR5	0.828 0.700 0.792 0.750 0.785	0.909	5.41	0.917
6	Perceived value	PV1 PV2 PV3 PV4	0.910 0.888 0.899 0.849	0.918	5.22	1.04
7	f-commerce usage intention	ITU1 ITU2 ITU3 ITU4	0.831 0.827 0.841 0.859	0.932	5.62	0.932

All factor loadings are significant at the *p* < 0.001 level.

Convergent validity was inspected by applying factor loadings, composite reliability (CR), and the average variance extracted (AVE). The result of CFA from Table 2 shows that all the loadings range from 0.70 to 0.91 and are significant at the *p* < 0.001 level. The result from Table 3 describes the outcomes of CR are greater than 0.9, and the AVE is greater than 0.6. These values are greater than the defined values, which implies higher convergence, as suggested by Fornell and Larcker (1981) and Hair et al. (1998, 2010).

**Table 3:** Inter-construct correlation.

	CR	AVE	DT	TR	TeR	ITU	PV	eWOM	SPA
DT	0.947	0.750	0.866						
TR	0.909	0.669	0.465	0.818					
TeR	0.915	0.684	0.428	0.522	0.827				
ITU	0.932	0.773	0.407	0.528	0.332	0.879			
PV	0.918	0.738	0.016	0.091	0.014	0.316	0.859		
eWOM	0.919	0.739	0.400	0.522	0.484	0.358	0.006	0.860	
SPA	0.912	0.721	0.382	0.503	0.448	0.351	0.036	0.423	0.849

Diagonal elements are the square root of the average variance extracted (AVE) from each construct; Pearson correlations are shown below the diagonal, *p* < 0.05.

Discriminant validity was gauged as per the description of Fornell and Larcker (1981), relating the inter-construct correlations and the square root of AVE. Table 3 explained that the values of the square root of AVE are higher than the correlation coefficients of all variables, which indicates good discriminant validity.

As per the good-fitness of the model is concerned, the values of CMIN/DF = 2.109, CFI = 0.957, SRMR = 0.031, RMSEA = 0.051, and PClose = 0.413 are under the cutoff criteria for fit indexes as proposed by Hu and Bentler (1999). The results describe that the model of this study has no issue regarding reliability and validity, suggesting the significant variation among the constructs that could be used for the structural model.

### Structural Model

**Figure 2:**
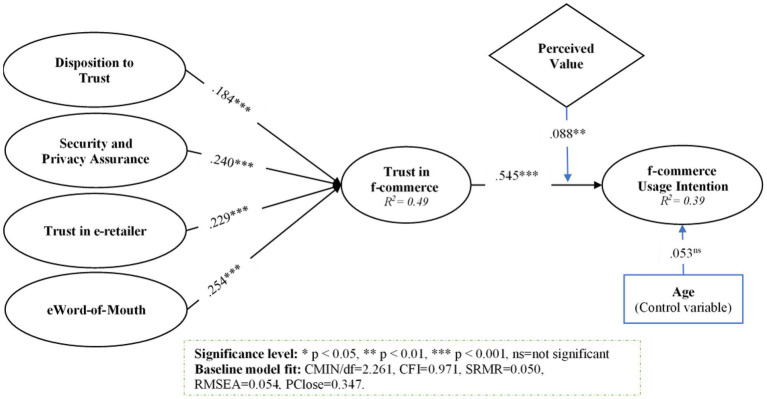
Results of structural model.

Subsequently, testing the reliability and validity of the research model, the hypothetical relationship between constructs was measured using AMOS v24. Figure 2 represents the values of the path coefficient; meanwhile, also revealed in Table 4. The values of R-square from Figure 2 describe that 49% variation in the trust in f-commerce is due to selected factors, while 39% variation has been explained in f-commerce usage intention (ITU).

**Table 4:** Structural equation modeling (SEM) results for hypotheses.

Hypotheses			Std. estimate	S.E.	C.R.	*p*	Remarks
TR	<---	DT	0.184	0.040	4.553	***	H1: Supported
TR	<---	SPA	0.240	0.041	5.811	***	H2: Supported
TR	<---	TeR	0.229	0.044	5.241	***	H3: Supported
TR	<---	eWOM	0.254	0.043	5.968	***	H4: Supported
ITU	<---	TR	0.545	0.037	14.600	***	H5: Supported
ITU	<---	PV*TR	0.088	0.035	2.356	**	H6: Supported
ITU	<---	Age	0.044	0.053	1.180	0.238	Not significant

Significance:

** *p* < 0.01

*** *p* < 0.001.

Based on results from SEM analysis, disposition to trust (*β* = 0.184, *p* < 0.001), security and privacy assurance (*β* = 0.240, *p* < 0.001), trust in e-retailer (*β* = 0.229, *p* < 0.001), and eWoM (*β* = 0.254, *p* < 0.001) exposed the positive variance in trust in f-commerce. Thus, H1, H2, H3, and H4 are supported based on these empirical findings. Moreover, trust substantially influences consumer f-commerce usage intention (*β* = 0.545, *p* < 0.001), supporting H5. The control variable’s value is not significant, showing no impact on f-commerce usage intention in the current study. Moreover, in Figure 2 the values of CMIN/DF = 2.261, CFI = 0.971, SRMR = 0.050, RMSEA = 0.054, and PClose = 0.347 are showing the good fitness of the baseline structural model.

### Moderating Role Perceived Value

This research also concerns the moderating impact of consumer perceived value regarding behavior intentions to use the f-commerce. Hypothesis 6 stated that perceived value moderates the link between trust and f-commerce usage intention. The output from Table 4 indicates interaction term (PV*TR), significantly and positively affect the relationship between trust in f-commerce and f-commerce usage intention (*β* = 0.88, *p* < 0.0), endorsing the hypothesis. Moreover, by using the stats tools package of Gaskin (2012), Figure 3 represents the result of two-way interaction, which confirmed that perceived value strengthens the positive relationship of trust in f-commerce and f-commerce usage intention.

**Figure 3:**
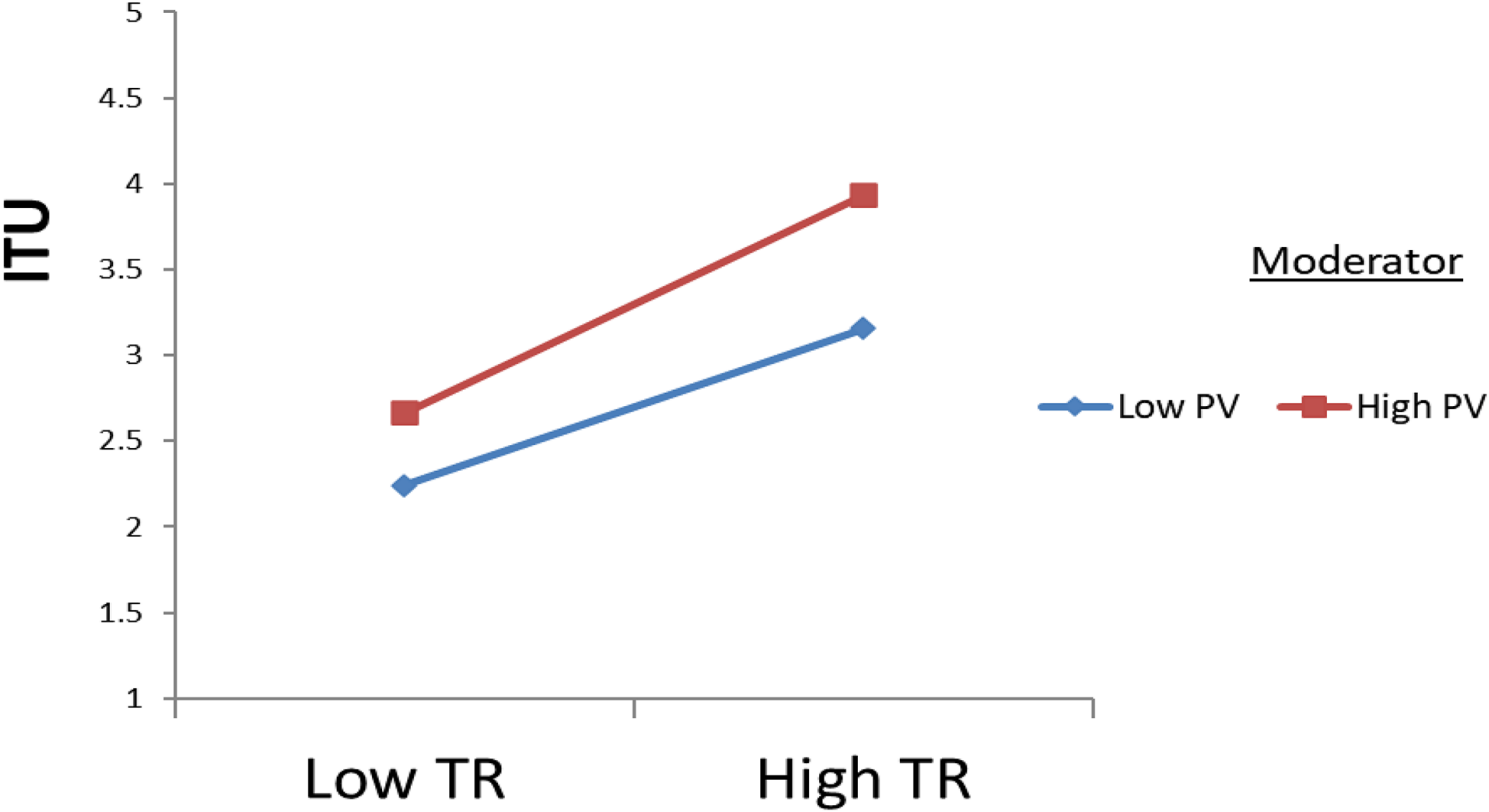
Moderating effect of PV.

### Moderating Effects of Gender

A multi-group test was performed to investigate the moderating impact of gender. Firstly, males and females are separately skirted into two groups from the entire dataset, and the multi-group test is executed. Table 5 demonstrates the results of gender differences. According to the results, gender moderates the relationship between disposition of trust and trust in f-commerce, security & privacy assurance and trust in f-commerce, trust in f-commerce, and f-commerce usage intentions, and moderating impact of perceived value between the relationship of trust in f-commerce and f-commerce usage intention. Furthermore, there are no gender differences between the relationship of trust in e-retailer and eWoM with trust in f-commerce. Because most women have built a long-term relationship of trust, their decisions are less flexible. They may not be able to readily switch their intentions from one person/entity to another, depending on their personality. There is a widespread belief that women are reluctant to adopt new technologies and slowly adopt new shopping habits (Ibrahim et al., 2017). Probably because of their indecisive character, women require greater trust in the precise pattern of purchasing and selling, which is why they reacted so strongly to the disposition of trust, security and privacy assurance, and trust in f-commerce compared to men.

**Table 5:** Multi-group analysis of gender as a moderator.

Hypotheses	Path coefficient results				
	Male	Female	Difference	Hypothesized effect	Remarks
TR <--- DT	−0.040^**^	0.252^***^	0.292^***^	Different (Male < Female)	H7a: Supported
TR <--- SPA	0.089	0.251^***^	0.162^*^	Different (Male < Female)	H7b: Supported
TR <--- TeR	0.134^*^	0.205^**^	0.071ns	No Difference (Male = Female)	H7c: Not supported
TR <--- eWOM	0.186^***^	0.119^*^	0.067ns	No Difference (Male = Female)	H7d: Not supported
ITU <--- TR	0.334^***^	0.587^***^	0.253^**^	Different (Male < Female)	H7e: Supported
ITU <--- PV*TR	−0.250^***^	0.120^*^	0.370^***^	Different (Male < Female)	H7f: Supported

ns = not significant. Significance: *p*-values.

* *p* < 0.05,

** *p* < 0.01, and

*** *p* < 0.001.

### Discussion

Disposition to trust is a person’s inherent tendency to trust or distrust others (Escobar-Rodríguez et al., 2017). Individuals’ propensity to trust does not vary with specific entities, but rather is a stable feature of their personality that governs how they view the trustworthiness of other entities they encounter (Mahad et al., 2018). The results of this study also described the positive impact of disposition to trust on the development of individual trust in f-commerce.

One of the key barriers to shopping online is the dearth of awareness about security and privacy concerns. Several incidents in which online merchants encounter security vulnerabilities or inadvertently leak consumers’ personal and credit card information can seriously damage consumer confidence (Shankar et al., 2002). Our results from this study also proved the significant positive influence of security and privacy assurance in building consumer trust in f-commerce. The e-retailers should reduce consumer privacy concerns and improve consumer security by adopting and sharing clear privacy policies, statements, and innovative payment technologies and processes. Therefore, advanced security and privacy methods need to be set up to improve consumers’ trust and willingness to use f-commerce. In line with these outcomes, the empirical results also proved that trust in e-retailer is also an important predictor in developing consumer trust in f-commerce. The absence of trust in e-retailers is bound to pay off by a high degree reduction of trust in f-commerce and vice versa. Subsequently, compared with traditional e-commerce, the buying decision is not solitary inclined by the trust acuity of the e-retailer but also inclined by the f-commerce platform.

Based on our empirical outcomes, it has been concluded that eWoM significantly influences creating and enhancing confidence in the consumers’ minds to use the f-commerce. eWoM was discovered by various studies to determine human behavior, expressly in the arena of e-commerce (Awad and Ragowsky, 2008; Liébana-Cabanillas and Alonso-Dos-Santos, 2017). The findings of this study also related to prior literature. Meanwhile, the sense of personal warmth, contact, and positive eWoM develop a stable environment for transactions permitted within the f-commerce and vice versa. It also confirms that trust can be shifted from identified reliable parties in social networks to unidentified parties that can sell social networks through eWoM. Our study results exposed that trust is a credible predictor of consumers’ willingness to use, and the antecedents of trust are particularly important in the f-commerce environment, along with the research of Brock et al. (2011) and Hajli et al. (2017). These are the important findings that are conducive to online trust research and point out the precise aspects affecting user trust in f-commerce.

Moreover, the interaction effect of perceived value remains also investigated in the association of trust in f-commerce and f-commerce usage intention. The results proved that perceived value has significantly moderated the relationship of trust in f-commerce and f-commerce usage intention among the citizen of Pakistan. Changes in behavioral intention related to the f-commerce platform for online shopping are important because trust leads to similar changes in perceived value. It means that a decline in consumer trust may turn to another platform that provides better value.

Similarly, empirical investigation of gender differences is an important contribution to f-commerce adoption, which was limited in previous literature on f-commerce. The findings proved that gender significantly moderates the relationships of trust, security, and privacy assurance with trust in f-commerce. Moreover, perceived value also has a moderating impact on the relationship of trust in f-commerce and f-commerce usage intention and moderating role of perceived value on the relationship between trust in f-commerce and f-commerce usage intention. In all these selected relationships, females are dominant compared to males, and our findings are consistent with the study of Gefen and Straub (2006) and Dutta et al. (2018). However, the outcomes regarding the dominance of females are fascinating in this study. The female dominance means that with increasing the disposition to trust and security and privacy assurance, trust in the female will increase more than males regarding f-commerce use intention. Furthermore, our results have demonstrated that as much trust in f-commerce will increase, f-commerce usage intention will rise more in females than in males. Similarly, the moderating role of perceived value among the relationship of trust in f-commerce and f-commerce usage intention is more effective in the case of females.

The reason behind these results is that most women have established long-term trust, and their decisions are less flexible. They might not easily shift their intentions from one person/entity to another. It is a general perception that females have a fear of adopting technology and mostly is laggard in the adoption of new patterns of buying (Ibrahim et al., 2017). Due to its uncertain nature, the female needs more trust regarding the precise pattern of buying and selling, and it might be the reason that females reacted so strongly as males regarding f-commerce usage when they got disposition of trust, security, and privacy assurance and trust on f-commerce. According to the study of Hsiao (2009), females are the more keen buyer, and value addition is more attractive in females as compared to males, especially in developing countries. It might be the reason the females are dominant in the moderating impact of perceived value in the association of trust in f-commerce and f-commerce usage intention. Therefore, the conclusions of our research are endorsed as well by past investigations. However, there are no gender differences among the relationships of eWOM; and trust in e-retailer with trust in f-commerce. Moreover, it is advised that online businesses eager to use the Facebook platform to sell products should develop strategies to enhance the trust in online communities by providing security and privacy assurance to increase consumers’ intentions to use f-commerce.

## Conclusion

Due to the high uncertainty linked with the use of the Facebook platform for shopping, the development and transfer of trust in f-commerce is the most important aspect to be described, particularly in the developing world. In the real world, organizations such as Starbucks and Ticketmaster have created highly successful f-commerce operations and utilization of f-commerce aspects in sales expansion. This research evaluated the series of relationships between antecedents of trust in f-commerce, which lead to f-commerce usage intention in Pakistan. The results confirm the significant positive association between the predictors of trust as well as with f-commerce usage intention. Based on the findings of SEM analysis, a disposition to trust has been identified significantly and positively linked with the trust in f-commerce and supported the study hypothesis. The study results show that in Pakistan, consumers’ disposition to trust plays an imperative part in creating individual trust in f-commerce, which ultimately leads to their increased intentions to use f-commerce. In addition, our research results show that females’ trust in f-commerce will increase, and their willingness to use f-commerce will be higher than that of men. Similarly, the moderating role of perceived value in the prescribed relationships is more effective for females. Moreover, theoretical, and practical implications are given based on empirical analysis.

### Theoretical Implications

The current paper pointedly contributes to the ongoing literature of f-commerce, specifically and social commerce, generally. First, the research covers the existing literature by investigating and confirming a research framework that incorporates the factors of trust, which was evidenced to be a key factor of f-commerce. Second, several studies describe the various aspects of trust factors in e-commerce, but, as far as we know, current research is among the first to hypothetically inspect the precursors of trust in the presence of consumer perceived value as moderating factor to measure the consumer trust in f-commerce which further leads toward the f-commerce usage intention.

Third, recent studies on user adoption intentions are inadequate to trust or reference factors, particularly in the settings of f-commerce. Conclusions contained in the report of the moderating impact of perceived value have a valuable theoretical contribution for the researchers since it was uncovered that consumer perceived value has a positive influence on the association of trust in f-commerce and usage intention of f-commerce. The perceived value that consumers derive from using the suggested platform and e-retailer is also considered the key aspect in determining their ultimate usage intention.

Finally, our results also contribute to the current literature by clarifying the gender differences in f-commerce adoption. In most cases, females were found to be more concerned about the factors of trust in f-commerce and their decision to f-commerce usage tendency. Put it another way, demographic variables might be able to moderate the underlying relationships between precursors of trust in f-commerce and usage intention. Therefore, when the trust is applied in other contexts, researchers can also examine the moderating effects.

### Practical Implications

Despite the industry’s confidence in the nascent of f-commerce, it was frequently futile. According to Beach (2015), in 2011, the top 101 brands sold products on Facebook, and in 2015, there were only 40. Online stores using the Facebook platform continually apply advanced technical tools to encourage f-commerce transactions with the high expectations of performing well. However, a substantial slice of consumers does not believe in f-commerce due to a lack of trust compared to other e-commerce platforms.

In line with prior literature, this study demonstrates that enlightening trust in the f-commerce platform is another essential dimension in its prospective development, so it is essential to reassure online vendors to explicate the benefits of shopping through Facebook, facilitate payment, return guidelines, and correct mistakes.

Online vendors from social network sites who are willing to trade using the f-commerce should build a hype of trust among potential consumers by assuring consumer security and privacy, which enhances their intentions to use the f-commerce platform that were outlined and investigated here. Since the study described that e-retailers could be trusted by improving their image, they can also improve the Facebook network’s image to encourage f-commerce in Pakistan and other developing countries.

In line with the above cases, it is suggested that companies who decide to involve in commercial activities using f-commerce must consider these factors to enhance consumer trust. Improved eWoM can enhance the trust and improve the consumer intentions to use; for this purpose, the company should focus on the initial discount policy, initiate help tutorial process, promotion, and price action. These policies enhance trust and raise the level of perceived value that ultimately contributes to the f-commerce usage intention. Furthermore, this study also guided the practitioners on which factor can affect more the behavior of females regarding the usage intentions of f-commerce that might help in the practical adoption of f-commerce and increase sales performance.

### Limitations and Future Research Guidelines

The authors concede few limitations. First, due to the cross-section design, this research confines the fortitude of causality. Future academics should pay further consideration to test and authenticate the outcomes of this research in a diverse cultural context using a longitudinal approach, which allows testing the strength of the relationship from a time perspective. Second, in this research, most participants belonged to the young age group. Increasing the popularity of online shopping, consumers of different ages have started using f-commerce. Hence, it is required to probe the level of trust influencing the usage intention about f-commerce at a different age group. Third, the authors used the survey method to investigate consumers intends to use f-commerce instead of actual user behavior. Although prior literature sources support solitary behavioral intentions as predictions of actual behavior, this is not always correct (Gefen et al., 2003; Crossler et al., 2013). Thus, future research may also study a method to acquire actual behavior regarding the use of f-commerce by integrating survey and interview methods. Finally, this study only discussed the direct positive impact of eWoM in developing trust in f-commerce. Future scholars are suggested to describe the valance of eWoM (such as positive, negative, the content of eWoM) in developing trust in f-commerce.

## Data Availability Statement

The raw data supporting the conclusions of this article will be made available by the authors, without undue reservation.

## Ethics Statement

The studies involving human participants were reviewed and approved by Department of Management Science, Islamia University of Bahawalpur, Bahawalnagar Campus, Pakistan. Written informed consent for participation was not required for this study in accordance with the national legislation and the institutional requirements.

## Author Contributions

FS: conceptualization, supervision, and writing—original draft preparation. FS and IK: methodology. IK and AS: software and data curation. IK: formal analysis. JW: resources, project administration, and funding acquisition. JW and IK: writing—review and editing. All authors contributed to the article and approved the submitted version.

## Funding

This research was funded by the Henan Education Department of Humanities and Social Sciences Research Project, Grant Number: 2022-ZZJH-420 and Key Program of Teaching Reform in Higher Education of Heilongjiang Province, Grant Number SJGZ20200148.

## Conflict of Interest

The authors declare that the research was conducted in the absence of any commercial or financial relationships that could be construed as a potential conflict of interest.

## Publisher’s Note

All claims expressed in this article are solely those of the authors and do not necessarily represent those of their affiliated organizations, or those of the publisher, the editors and the reviewers. Any product that may be evaluated in this article, or claim that may be made by its manufacturer, is not guaranteed or endorsed by the publisher.

## Appendix

**Appendix A:** Measurements items of the constructs.

Variables	Items		Sources
Disposition to trust	DT1	“I generally trust other people on the Facebook platform.”	Han and Windsor, 2011; Sharma et al., 2019
	DT2	“I tend to count upon other people on the Facebook platform.”	
	DT3	“I generally have faith in humanity.”	
	DT4	“I feel that people are generally reliable.”	
	DT5	“I generally trust other people unless they give a reason not to do.”	
	DT6	“I feel the f-commerce user’s recommendations are mostly honest.”	
Security and privacy assurance	SPA1	“I believe that shopping on Facebook is reliable.”	Vijayasarathy, 2004; Brock et al., 2011
	SPA2	“Facebook shopping cannot be trusted; there are too many uncertainties.” (Reverse coded)	
	SPA3	“I cannot rely on Facebook vendors to keep the promises that they make regarding the security and privacy of personal information.” (Reverse coded)	
	SPA4	“In general, making payments on the f-commerce platform is secure.”	
Trust in e-retailer	TeR1	“I believe that the e-retailer would act in my best interest.”	Brock et al., 2011
	TeR2	“I believe that e-retailer would do their best to help me when I need it.”	
	TeR3	“I believe that the e-retailer on the f-commerce platform would keep its commitments.”	
	TeR4	“The e-retailer has sufficient expertise and resources to do business on the Internet.”	
	TeR5	“I believe that e-retailer is sincere about the delivery of good products and services on the f-commerce platform.”	
e-Word of mouth	eWoM1	“In using f-commerce, I generally buy brands I think other people will approve of.”	Chu and Kim, 2011; Liébana-Cabanillas and Alonso-Dos-Santos, 2017
	eWoM2	“I prefer to take opinions about products and retailers on Facebook before buying a product.”	
	eWoM3	If other people can see me using a product, I frequently buy the brand they expect me to buy.”	
	eWoM4	“I find a sense of belonging by purchasing the same products and brands that others buy.”	
Trust in f-commerce	T1	“I believe that the f-commerce platform will keep the promises and commitments they make.”	Brock et al., 2011; Liébana-Cabanillas and Alonso-Dos-Santos, 2017
	T2	“I believe that the information offered by the f-commerce platform is sincere and honest.”	
	T3	“I believe that the f-commerce platform is responsible.”	
	T4	“I think f-commerce has the necessary resources to carry out its activities successfully.”	
	T5	“I think I can have confidence in the promises that the f-commerce platforms make.”	
Perceived value	PV1	“The time it would take to make purchases on f-commerce platforms is very reasonable.”	Sweeney and Soutar, 2001; Liébana-Cabanillas and Alonso-Dos-Santos, 2017
	PV2	“The effort associated with the use of the Facebook platform to make online purchases is worth it to me.”	
	PV3	“The perceived experience in the use of f-commerce is positive.”	
	PV4	“I believe that the use of f-commerce platforms to be valuable.”	
f-commerce usage intention	ITU1	“I intend to use a Facebook platform for purchasing a product or service in the future.”	Venkatesh and Davis, 2000; Sharma et al., 2019
	ITU2	“I believe using f-commerce is very convenient to buy products.”	
	ITU3	“I would recommend others to use an f-commerce for their next purchase.”	
	ITU4	“I prefer to use f-commerce as an f-commerce on a regular basis.”	
